# What is life?

**DOI:** 10.1007/s11033-021-06594-5

**Published:** 2021-07-27

**Authors:** Jaime Gómez-Márquez

**Affiliations:** Department of Biochemistry and Molecular Biology, Faculty of Biology - CIBUS, University of Santiago de Compostela, 15782 Santiago de Compostela, Galicia Spain

**Keywords:** Life definition, Living viruses, Robots, Extraterrestrial life

## Abstract

**Background:**

Many traditional biological concepts continue to be debated by biologists, scientists and philosophers of science. The specific objective of this brief reflection is to offer an alternative vision to the definition of life taking as a starting point the traits common to all living beings.

**Results and Conclusions:**

Thus, I define life as a process that takes place in highly organized organic structures and is characterized by being preprogrammed, interactive, adaptative and evolutionary. If life is the process, living beings are the system in which this process takes place. I also wonder whether viruses can be considered living things or not. Taking as a starting point my definition of life and, of course, on what others have thought about it, I am in favor of considering viruses as living beings. I base this conclusion on the fact that viruses satisfy all the vital characteristics common to all living things and on the role they have played in the evolution of species. Finally, I argue that if there were life elsewhere in the universe, it would be very similar to what we know on this planet because the laws of physics and the composition of matter are universal and because of the principle of the inexorability of life.

## Introduction

Life is a wonderful natural process that occurs in highly organized dynamic structures that we call living beings. Today, thanks to the enormous advance of Biology, we know and understand much better the vital phenomenon, the molecular biology of the cells, the enormous biodiversity on our planet, the evolutionary process, and the complexity of ecosystems. However, despite these enormous advances, biology still lacks a solid theoretical framework necessary to understand the vital phenomenon and to answer questions such as what is life? or are viruses living entities? To answer these and other fundamental questions related to life, in addition to the universal laws of physics, biology needs its own principles to help us find answers to major theoretical challenges such as the origin of life, the construction and maintenance of genomes, or the concept of life itself. Regarding the principles governing life, there have been several contributions from different perspectives (e.g. [1–5]) and I myself have proposed a series of principles (named as the commandments of life) to explain and understand the vital phenomenon from an evolutionary perspective, far from any vitalist, pseudo-scientific or supernatural considerations [6].

In the words of B. Clark, a definition of life is needed more than ever before to provide defendable objective criteria for searches for life on other planets, to recognize critical distinctions between machine life and robots, to provide insight into laboratory approaches to creating test-tube life, to understand the profound changes that occurred during the origin of life, and to clarify the central process of the discipline of biology [7]. It is worth noting what E. Koonin wrote about the complexity of defining life: “In my view, although life definitions are metaphysical rather than strictly scientific propositions, they are far from being pointless and have potential to yield genuine biological insights” [8]. However, despite its importance there is no widely accepted definition of what life is and some of the most commonly employed definitions (see below) face problems, often in the form of robust counter-examples [5, 9]. Even some scientists and philosophers of science suggest that it is not possible to define life [5, 8].

## What is life?

We can define life in very different ways depending on the context and the focus we want to give to the definition. For example, we can define life as the period from birth to death or as the condition that occurs only in living organisms. We can also say that life is a wonderful and ever-changing process that occurs in highly organized receptacles that we identify as living entities. Likewise, the popular encyclopedia Wikipedia define life as “a characteristic that distinguishes physical entities that have biological processes ….. from those that do not …” [10]. However, with these expressions we are not defining precisely what life is and therefore we need to create a definition that concisely but informatively reflects our scientific knowledge of the vital phenomenon. We have to distinguish between life and living matter, which is the place where life lives, and between living beings and non-living matter. In reality, when we ask ourselves “what is life?” we are asking “what are the characteristics that distinguish a living organism from a non-living entity?

There are numerous definitions of life formulated from different characteristics of living beings (replication, metabolism, evolution, energy, autopoiesis, etc.) and from different approaches (thermodynamic, chemical, philosophical, evolutionary, etc.). Often, definitions of life are biased by the research focus of the person making the definition; as a result, people studying different aspects of biology, physics, chemistry, or philosophy will draw the line between life and non-life at different positions [11]. These strategies create a panoply of alternative definitions that makes it very difficult to reach a consensus on the best definition of life because they all have pros and cons. [12, 13]. Let me briefly discuss some of the most representative definitions of life. There is a short definition “Life is self-reproduction with variations” [14] that is interesting for its brevity and because it includes two fundamental characteristics of living organisms: reproduction and evolution. However, this minimalist definition is clearly insufficient [8] and it does not include some of the most important traits we see in living things. Along these lines, there is also the definition coined by NASA: “Life is a self-sustaining chemical system capable of Darwinian evolution” [15, 16]. This is a more complete and, I believe, better definition than the previous one, as it incorporates, in addition to reproduction and evolution, metabolism. However, both definitions are unsatisfactory because neither cell nor multicellular organisms are self-sufficient as there is always a dependence on other organisms and external factors to live and reproduce. Furthermore, these definitions say nothing about the chemical nature of living matter, the interactions with the environment or the low entropy of living things. Apart from that, reproduction being essential for the perpetuation of species and evolution, not all living beings are able to reproduce (e.g. the mule, most bees, etc.) and do not thereby lose their living status. A more recent definition of life states: “Life is a self-sufficient chemical system far from equilibrium, capable of processing, transforming and accumulating information acquired from the environment” [17]. Although this definition is more comprehensive than the previous ones and includes a reference to thermodynamics, in my opinion it has four drawbacks: (i) the term “self-sufficient” is not adequate because the quality of life does not provide self-sufficiency; (ii) the thermodynamic component does not highlight how fundamental low entropy or high order is for any living being; (iii) information can be acquired from “within” and not only from the environment; (iv) life is not a system is a process and living beings are the system where that process occurs (I discuss this point below). From a very different perspective it was defined life as “matter with the configuration of an operator, and that possesses a complexity equal to, or even higher than the cellular operator” [18]. This proposal introduces a new term, the operator, which is somewhat confusing, excludes viruses and makes a strange classification of living systems. On the other hand, some scientists have also attempted to define life from a handful of key features. Thus, seven “pillars” (the essential principles by which a living system functions) have been proposed on which life as we know it can be defined [19], but no definition was provided. Life has also been considered as any system that from its own inherent set of biological instructions and the algorithmic processing of that "prescriptive information" can perform the nine biofunctions [20] which are basically the same as the "pillars" mentioned above. However, no definition of life was proposed, and again it was considered as a system rather than a process. Both definitions exclude viruses as living beings, mainly because the existence of a membrane, a metabolic network and self-replication are set as conditions for life. In short, there are many more definitions of life but as R. Popa says “We may never agree on a definition of life, which will remain forever subject to a personal perspective” [21].

## My definition of life

Traits are measurable attributes or characteristics of organisms and trait-based approaches have been widely used in systematics and evolutionary studies [22]. Since any definition of life must connect with what we observe in nature, my strategy for finding a definition of life was to establish what are the key attributes or traits common to all living things. What do bacteria, yeasts, lichens, trees, beetles, birds, whales, etc. have in common that clearly differentiates them from non-living systems? In my opinion, living organisms share seven traits: organic nature, high degree of organization, pre-programming, interaction (or collaboration), adaptation, reproduction and evolution, the last two being facultative as they are not present in all living beings.

Organic nature and highly organized structures. Living matter is organic because it is based on carbon chemistry and molecular interactions take place following the laws of chemistry. As R. Hazen wrote “Carbon chemistry pervades our lives. Almost every object we see, every material good we buy, every bite of food we consume, is based on element six. Every activity is influenced by carbon—work and sports, sleeping and waking, birthing and dying.” [23]. Living organisms are highly organized structures that maintain low entropy (the vital order) by generating greater disorder in the environment, thus fulfilling the postulates of thermodynamics [24, 25]; when this vital order is lost, life disappears and the only way to restore life is to generate a new vital organized structure through reproduction [6]. Living organisms resist entropy thanks to biochemical processes that transform the energy they obtain from nutrients, sun or redox reactions. It could be said that vital order and energy are two sides of the same coin.

Pre-programming. Every living entity has a software (a pre-programme) in its genetic material that contains the instruction manual necessary for both its construction (morphology) and its functioning (physiology). This programme has been modified in the course of evolution, as a consequence of contingency and causality, so it is not a static or immutable program but a dynamic one. Furthermore, there is also another preestablished *program* that conditions the vital phenomenon and that I have called the principle of inexorability [6]. Let me give few examples of the principle of inexorability at different levels of complexity. The shape of ribosome is determined (pre-programmed) by the chemical bonds that are established between ribosomal proteins and rRNA. A similar example is the λ phage morphogenesis that depends only on interactions protein–protein and protein-DNA. Evolutionary convergence or the need for wings to fly are other examples of this inexorability guided by the laws of nature.

Interaction and adaptation. If we look at nature in its purest state or at the complex human society, we can see countless interactions between living beings and with their environment necessary for survival and reproduction. We can see interactions at the molecular level (e.g., allosteric interactions, metabolic pathways, cellular signaling, quorum sensing), in the relationships between organisms of the same or different species (e.g., sexual reproduction, symbiosis, infection, parasitism, predator–prey, or sound language), or between living forms and the environment (e.g., photosynthesis or physiological/anatomical interactions for swimming or flying). Interaction is collaboration, it is cooperation at all levels [6], the ecosystem being the best example of multiple collaborative interactions between very different organisms. In terms of adaptation, living organisms show a great capacity to adapt both to their surroundings and to environmental circumstances; furthermore, adaptations involving new biological characteristics can be seen as an opportunity to find a different way to evolve. In this sense, the evolutionary process reflects this continuous adaptation and anatomy, physiology and genome bear witness to this. Life is adaptative because species adapt to environmental changes modifying their physiology or metabolism, for instance reducing heartbeat during hibernation (e.g. the grizzly bear *Ursus arctos horribilis*) or synthesizing fat from excess sugar to increase the energy reserves of the body (e.g. *Homo sapiens*). In addition to these temporal adaptations in response to environmental changes [26], there are also changes in genotype or phenotype since the adaptation process is the result of natural selection acting upon heritable variations [27]; a well-known example of this is the peppered moth *Biston betularia* whose allele frequencies of the locus that controls the distribution of melanin in the wings changed with the industrial revolution in England [28]. Epigenetic variations also contribute to rapid adaptative responses [29, 30].

Reproduction and Evolution. Another property of living beings is their ability to perpetuate themselves and thus make it possible for the species not to disappear and to evolve. Reproduction can be observed at the molecular (DNA replication), cellular (mitosis, meiosis, binary division), and organismal (sexual and asexual) levels. From a different perspective, reproduction is also the way to overcome the second law of thermodynamics and the tyranny of time because when we reproduce, we are creating a new order and resetting the vital clock to zero [6]. What about individuals such as the mule or the male and female of a species, or the hermaphrodite that cannot self-fertilize, who cannot reproduce because they are sterile or because they need another member of their species to reproduce? Are not these organisms living beings? Of course, they are! In this context, reproduction must be considered as a facultative trait because not all living organisms are fertile or can produce offspring on their own but maintain all other traits necessary for the life process. If an individual is sterile, the species will continue to exist because the evolutionary process must be analyzed at the population level, not at the level of individual organisms; obviously, if the entire population were sterile, then the species would disappear and there would be no life. All species have the capacity to evolve, and this property is unique to life. Evolution allows living beings to adapt to new circumstances and the best genomes are selected and transmitted to the next generations. The concept of evolution (reproduction with variations and permanence in time) allows us to interpret the reality of the life we observe now and to guess what it has been like in the past. We cannot predict the future because evolution is not a finalistic process, it is, to use the words of J. Monod, the fruit of chance and necessity.

There is nothing on this planet, apart from a living being, that complies with all these characteristic features of living beings. It should therefore be possible to define life by logically combining them. Consequently, I define life as a process that takes place in highly organized organic structures and is characterized by being preprogrammed, interactive, adaptative and evolutionary. If life is the process, a living organism is the system in which that process takes place and which is characterized as organic, highly organized, pre-programmed, interactive, adaptative, and evolutionary. Why do I say that life is a process and not a system? According to the Merriam-Webster dictionary, a process is a natural phenomenon characterized by gradual changes that lead towards a certain result. A second meaning defines it as a continuous natural or biological activity or function; and a third one as a series of actions or operations conducing to an end. These three meanings of what a process is fit very well with what we observe happening in living beings, which is none other than the vital process or life. The dictionary itself defines a system as a regularly interacting or interdependent group of items forming a unified whole, and as an assemblage of substances that is in or tends to equilibrium or a group of body organs that together perform one or more vital functions. Once again, these definitions fit very well with what a living being represents.

What is the difference between life, living being and a robot? [31] Life is the vital process and the living being is the system, the “container” in a metaphorical way, where the vital process takes place. Following this reasoning, a robot would be an artificially organized, pre-programmed and interactive system, but unlike a living being it is not alive because it is neither organic, nor does it reproduce, adapt, or evolve. A robot or a population of robots cannot “reproduce and evolve” on its own, without the intervention of its "creator" (the human being), it will always need to be built or programmed by an engineer to do so. I do not dispute that the robot can adapt, especially thanks to advances in artificial intelligence, although I am not sure that it can do so in the biological sense of the term. Biological adaptation is a process by which a species eventually adapts to its environment as a result of the action of natural selection on phenotypic characteristics [32]. A robot may be able to adapt to its environment, but what it cannot do is adapt itself through a selective process (without intervention from its creator) and change into a new type of robot (evolve). On the other hand, regarding the synthetic lifeforms named as *xenobots* [33], I think they cannot be considered as pure robots, but as an interface between living beings and artificial robots, as they are made from cells. In the future we will probably build robots so perfect that we can consider them as almost living beings and as the result of the intervention of a creator (their engineer), something that we cannot say about living beings unless we are creationists.

## Are viruses alive?

A. Turing, one of the pioneers in the development of computer sciences, wrote: “Can machines think? This should begin with definitions of the meaning of the terms “machine” and “think” [34]. To paraphrase Turing, we could ask ourselves: can viruses be considered living entities? And the answer to this question, so important for biology and still controversial [35], is to define what a virus is and what life is. At least from a theoretical point of view, biology should seek a clear and definitive answer to this question instead of adopting a skeptical attitude and assuming what K. Smith wrote in his classic book on viruses, “As to the question asked most frequently of all, are viruses living organisms? that must be left to the questioner himself to answer” [36].

Viruses are entities that straddle the boundary between living and non-living and therefore their biological status is controversial. A virus can be defined as an acellular infectious agent whose structure consists of a macromolecular complex of proteins and nucleic acids. Viruses are not cells, they do not metabolize substances, nor can they reproduce by themselves, grow, or breathe. Yet, regardless of whether we consider viruses to be living beings or not, they are an inescapable part of life and there is an undeniable biological connection between the virus and the organism it infects. Given the close interconnection between viruses and their hosts, it seems plausible that viruses play essential roles in their hosts [37]. For example, endogenous retroviral elements have shaped vertebrate genome evolution, not only by acting as genetic parasites, but also by introducing useful genetic novelty [38]. More recently, it was found in the human genome a gene regulatory network based on endogenous retrovirus that is important for brain development [39] and a new tamed retroviral envelope that is produced by the fetus and then shed in the blood of the mother during pregnancy [40].

Viruses are capsid-encoding particles that infect all kind of cells and share hallmark genes with capsidless selfish genetic elements, such as plasmids and transposons [41]. Traditionally, they have been regarded as lifeless agents because they have no metabolism of their own and need a cell to replicate and generate new viruses [42]. However, while this is true, I believe that this is not a definitive criterion for excluding them from the tree of life (more on this below). There are scientists in the opposite side that consider viruses as living beings that can evolve [43] and classify them as capsid-encoding organisms as opposed to the ribosome-encoding organisms that include all cellular life forms [37, 44]. Viruses have played a key role in the evolution of species [35] because they are the most abundant source of genetic material on Earth, are ubiquitous in all environments, and have actively participated in the exchange of genes or DNA fragments with their hosts [41, 45, 46].

We cannot say whether a virus is a living thing or not without defining what is life and what is a living thing. Obviously, if we take the cell as the minimum vital unit, we cannot consider viruses as living entities, and any discussion of this is superfluous. As far as I am concerned, considering viruses as non-living creatures because they need a cell to reproduce is not a very strong argument for two reasons. First, viruses are obligate intracellular parasites, and, like all parasites, they use the host for their own benefit, and this is their survival strategy. Viruses need nothing else to pursue the same goal as all species on this planet, which is to generate more viruses better adapted to infect new organisms. They apply the “law of least effort” to achieve this goal and may even *decide* to remain inside the host cell in a lysogenic manner, as in the case of bacteriophage lambda [47], or by establishing latency as herpesviruses do [48]. Second, as I said before no cell or organism is self-sufficient, as it needs at least a supply of food/energy to survive and reproduce. We know that life is absolutely interdependent. For example, we depend directly on our intestinal bacterial flora for our survival, and indirectly on nitrogen-fixing bacteria or photosynthesis. We could take to absurdity the argument that because viruses need a cell to reproduce, they are not alive and say that a man or a woman is not a living being because they cannot reproduce by themselves. The argument that a virus is not a living thing because it is an inert entity outside the cell is also not valid because such a virus could still have the ability to infect cells. Similarly, a spore or a seed cannot be considered lifeless because it is inert, as it is only waiting for the right environmental conditions to germinate, and that wait can last for thousands of years.

To answer the question of whether viruses are alive or not, I base my argument in support of considering viruses as living entities obviously on my own definition of life (this paper), as well as on what we know about the biology of viruses. First, viruses, like all cellular entities in nature, are composed of organic molecules; a virus consists of a nucleic acid (DNA or RNA), which is its genetic material as in all living things, and a protein capsid encoded by the viral genome that protects the viral genetic material and participates in the propagation of the virus in the host; viral capsids show fascinating dynamics during the viral life cycle [49]. Secondly, viruses are highly organized structures. There is an astonishing diversity of organization and geometric design of viruses, requiring only a few different structural subunits of the capsid to construct an infectious particle. Many viruses have developed very successful self-assembly systems; so much so that the viral capsid can self-assemble even outside the host cell [50]. The third feature common to all living things is that they are pre-programmed, and viruses also fulfill this characteristic because in their genetic material are written the instructions to make new viruses capable of infecting new cells or organisms. Viruses in their genome have the necessary (though not sufficient because they need elements provided by the host cell) instructions to make new viruses, and in this they are the same as any other living thing. In addition, the process of self-assembly to generate new viruses occurs spontaneously because the instructions to do it autonomously are both in the capsid-forming molecules themselves and in the nucleic acid, either DNA or RNA [49].

Two other characteristics of living organisms are the ability to interact with other living organisms (interaction) and to adapt genetically to new circumstances (adaptation). Viruses interact with their host in multiple ways: during infection, when their genes are expressed and their genome replicated, when virions are formed, when they integrate into the genome of the host cell, or when they engage in horizontal gene transfer processes. Viruses not only interact with their host, but also adapt by generating new variants that increase their ability to infect other cells, or by taking control of cell metabolism for their own benefit, or even to escape the immune response [51]. In terms of reproduction and evolution, which are two closely related processes, viruses reproduce in the host cell and evolve through changes in their genome. Viral evolution, like that of all living things, refers to the heritable genetic changes that a virus accumulates during its life cycle, which may arise from adaptations in response to environmental changes or host immune response. Because of their short generation times and large population sizes, viruses can evolve rapidly [52].

Microbiologist and Nobel laureate J. Lederberg said that “The very essence of the virus is its fundamental entanglement with the genetic and metabolic machinery of the host”. As far as I am concerned, this statement is essentially true and its profound meaning is, at least for me, further proof that viruses are living things. Viruses form part of many integrated biological systems, and they played an important role in the evolution of species [53]. They can exchange genetic material and participate in horizontal gene transfer [43] even between individuals from different species [54]. Due to their high frequency of mutation [55], viruses are so abundant in nature and present such a high degree of diversity that they constitute by themselves the virosphere [46]. This great viral biodiversity is proof that these living entities perform fundamental evolutionary and ecological functions [56, 57]. In conclusion, I believe that viruses should be considered as living entities that can participate in events as diverse as causing pandemics, destroying bacteria, causing cancer, or participating in horizontal gene transfer.

Following the metaphor of the “container” as the vessel or system (the living being) in which the life process takes place, the fact that viruses are obligated intracellular parasites and do not have a cellular structure and metabolism of their own does not seem to fit this metaphor. It is obvious that the virus cannot be the “container” where the life process takes place, since the virus, when outside the cell, is in a “dormant” state waiting to find a suitable host to infect and complete its life cycle; we could say that it is inert but not yet dead. Therefore, in the special case of viruses, the “container” is the cell. Once the virus finds its specific “container”, it can then reproduce, or integrate into the genome of the host cell, or remain as an episome, or intervene in the evolutionary process through exchange of genetic material. From genomic and metagenomic data, we know that co-evolution between viral and host genomes involves frequent horizontal gene transfer and the occasional co-option of novel functions over evolutionary time. We can say that viruses and their cellular hosts are ecologically and evolutionary intertwined [58].

I would like to refer to an interesting reflection on the defining characteristics of life and how viruses fit into this conceptual framework [59]. Thus, Dupré and O'Malley consider collaboration as a common criterion of life and I can only agree with this assessment; in this sense, in a previous paper on the principles that govern life [6], I use the expression “cooperative thrust” to refer to the importance of collaboration in the origin and evolution of living beings. Without considering collaboration or cooperation as a key interaction, we could not explain endosymbiosis, eukaryogenesis, metabolism, multicellularity, etc. In the present paper, collaboration is implicit in what I call interaction as a common and fundamental feature of all living things. Interestingly, these authors point out that “leaving viruses out of evolutionary, ecological, physiological or conceptual studies of living entities, would allow only an incomplete understanding of life at any level” [59]. Considering this emphasis on collaboration as a *sine qua non* condition for life, how does the world of viruses fit in? Dupré and O'Malley propose, and I agree, that viruses can be understood as alive when they actively collaborate (I mean when they are infecting the target cell) and when they do not collaborate (I would say they are inactive), they have at most a potential for life.

Finally, I would like to add that I am aware that there are many scientists who consider that viruses are not living beings basically because they do not have a cellular structure with all that this means. Therefore, this biological dilemma will probably be with us for a long time to come. I think it will only be resolved when we reach a consensus on what life is because only then will we be able to say categorically whether something is alive or not. This is what I have modestly tried to do in this paper.

## What would life be like elsewhere in the universe?

The massive number of exoplanets strongly suggests that there is a high probability that life evolved elsewhere in the universe. Astrobiologists are committed to the search for life in the cosmos and for that purpose it is very convenient to have a criterion about what life is [16]. How can we be sure that there is life on a distant planet? To do so, we need to define some biosignatures that can establish the possible existence of living things elsewhere in the universe [60], otherwise what are we looking for? In addition to this, it would also help a lot in this search for life on other planets, finding out how life began on Earth.

Some scientists and philosophers of science think that this preconception of what life is may be a problem rather than a solution in the search for life in other planets. C. Cleland in her book about the nature of life states, “Life is not the sort of thing that can be successfully defined. In truth, a definition of life is more likely to hinder than facilitate the discovery of novel forms of life” [5]. I do not entirely agree with this double statement because although we must be open-minded in the search for life outside our galactic home, at the same time I think it is a good idea to have a hypothesis based on the only certainty we have about vital phenomena, which is life on Earth, that will help in the design of the search for extraterrestrial life.

Is there life elsewhere in the universe? We don't know yet and it is probably only a matter of time before we find life on other planets or aliens find us. In my opinion if there is life elsewhere in the universe, it will most likely be similar to what existed, exists or will exist on our planet. Let us see why. First of all, the laws of physics and chemistry are universal and these laws, directly or indirectly, govern everything that happens with the matter of the universe. According to the cosmological principle, the same physical laws and models that applies here on Earth also works in all parts of the universe [61]; it is also assumed that physical constants (gravitational constant, speed of light, etc.) remain the same everywhere in the universe. Second, the elements that make up the matter of the stars are the same everywhere in the universe although in different proportions; the “periodic table” is the same for the whole universe. Whether life exists elsewhere in the universe based on a chemistry other than carbon we do not know and can only speculate, but what we do know for sure is that life on Earth is based on carbon chemistry, perhaps because it cannot be otherwise. Third, there is the aforementioned principle of inexorability [6]. In this context, what does this principle mean? It means that if the environmental conditions are suitable, glucose will be converted into pyruvate in an aqueous medium, chemiosmotic processes will be an important mechanism for generating chemical energy, flying organisms will have wings, or genetic information will be encoded in a language analogous or identical to what we know on Earth. According to this, the differences between the Earth living forms and the “space creatures” could be attributed to a different evolutionary stage or to specific environmental conditions. This hypothetical premise could be very important when developing projects that seek life elsewhere in the universe.

## References

[1] Dhar PK, Giuliani A (2010). Laws of biology: why so few?. Syst Synth Biol.

[2] Lane N (2015). The vital question.

[3] Shou W, Bergstrom CT, Chakraborty AK, Skinner FK (2015). Theory, models and biology. eLife.

[4] Cockell CS (2017). The laws of life. Phys Today.

[5] Cleland CE (2019). The quest for a universal theory of life.

[6] Gómez-Márquez J (2020). What are the principles that govern life?. Commun Integr Biol.

[7] Clark B, Kolb VM (2019). A generalized and universalized definition of life applicable to extraterrestrial environments. Handbook of astrobiology.

[8] Koonin EV (2012). Defining life: an exercise in semantics or a route to biological insights?. J Biomol Struct Dyn.

[9] Cleland CE, Chyba CF (2002). Defining life. Orig Life Evol Biosph.

[10] Wikipedia (2021) Life. Wikipedia, the free encyclopedia.

[11] Szostak J (2012). Attempts to define life do not help to understand the origin of life. J Biomol Struct Dyn.

[12] Luisi PL (1998). About various definitions of life. Orig Life Evol Biosph.

[13] Popa R (2003). Between necessity and probability: searching for the definition and the origin of life.

[14] Trifonov EN (2011). Vocabulary of definitions of life suggests a definition. J Biomol Struct Dyn.

[15] Joyce GF, Deamer DW, Fleischaker GR, Sudbury MA (1994). Forward. Origins of life: the central concepts.

[16] Benner SA (2010). Defining life. Astrobiology.

[17] Vitas M, Dobovišek A (2019). Towards a general definition of life. Orig Life Evol Biosph.

[18] Jagersop Akkerhuis GA (2010). Towards a hierarchical definition of life, the organism, and death. Found Sci.

[19] Koshland D (2002). The seven pillars of life. Science.

[20] Abel DL (2012). Is life unique?. Life.

[21] Popa R (2012). Merits and caveats of using a vocabulary approach to define life. J Biomol Struct Dyn.

[22] Gallagher R, Falster DS, Maitner BS (2020). Open science principles for accelerating trait-based science across the Tree of Life. Nat Ecol Evol.

[23] Hazen RH (2019). Symphony in C: carbon and the evolution of (almost) everything.

[24] Schneider E, Sagan D (2005). Into the cool: energy flow, thermodynamics, and life.

[25] Peterson J (2012). Understanding the thermodynamics of biological order. Am Biol Teach.

[26] Somero GN, Lockwood BL, Tomanek L (2017). Biochemical Adaptation: response to environmental challenges from life’s origins to the Anthropocene.

[27] Gardner A (2009). Adaptation as organism design. Biol Lett.

[28] Mueller L, Mueller L (2020). 1973 The evolution of melanism. Conceptual breakthroughs in evolutionary ecology.

[29] Schmid MW, Heichinger C, Coman Schmid D (2018). Contribution of epigenetic variation to adaptation in *Arabidopsis*. Nat Commun.

[30] Gómez-Schiavon M, Buchler NE (2019). Epigenetic switching as a strategy for quick adaptation while attenuating biochemical noise. PLoS Comput Biol.

[31] Bongard J, Levin M (2021). Living things are not (20th century) machines: updating mechanism metaphors in light of the modern science of machine behavior. Front Ecol Evol.

[32] Bock WJ (1980). The definition and recognition of biological adaptation. Am Zool.

[33] Kriegman S, Blackiston D, Levin M, Bongard J (2020). A scalable pipeline for designing reconfigurable organisms. Proc Natl Acad Sci USA.

[34] Turing AM (1950). Computing machinery and intelligence. Mind.

[35] Koonin EV, Starokadomskyy P (2016). Are viruses alive? The replicator paradigm sheds decisive light on an old misguided question. Stud Hist Philos Biol Biomed Sci.

[36] Smith K (1962). Viruses.

[37] Dupré J, Guttinger S (2016). Viruses as living processes. Stud Hist Philos Biol Biomed Sci.

[38] Katzourakis A, Gifford RJ (2010). Endogenous viral elements in animal genomes. PLoS Genet.

[39] Brattas L, Jönsson ME, Fasching L (2017). TRIM28 controls a gene regulatory network based on endogenous retroviruses in human neural progenitor cells. Cell Rep.

[40] Heidmann O, Béguin A, Paternina J, Berthier R, Deloger M, Bawa O, Heidmann T (2017). HEMO, an ancestral endogenous retroviral envelope protein shed in the blood of pregnant women and expressed in pluripotent stem cells and tumors. Proc Natl Acad Sci USA.

[41] Koonin EV, Dolja VV (2014). Virus world as an evolutionary network of viruses and capsidless selfish elements. Microbiol Mol Biol Rev.

[42] Moreira D, López-García P (2009). Ten reasons to exclude viruses from the tree of life. Nat Rev Microbiol.

[43] Forterre P (2010). Defining life: the virus viewpoint. Orig Life Evol Biosph.

[44] Raoult D, Forterre P (2008). Redefining viruses: lessons from mimivirus. Nat Rev Microbiol.

[45] Gilbert C, Cordaux R (2017). Viruses as vectors of horizontal transfer of genetic material in eukaryotes. Curr Opin Virol.

[46] Zhang Y-Z, Shi M, Holmes EC (2018). Using metagenomics to characterize and expanding virosphere. Cell.

[47] Casjens SR, Hendrix RW (2015). Bacteriophage lambda: early pioneer and still relevant. Virology.

[48] Nicoll MP, Proença JT, Efstathiou S (2012). The molecular basis of herpes virus latency. FEMS Microbiol Rev.

[49] Bruinsma RF, Wuite GJL, Roos WH (2021). Physics of viral dynamics. Nat Rev Phys.

[50] Baschek J, Klein H, Schwarz US (2012). Stochastic dynamics of virus capsid formation: direct versus hierarchical self-assembly. BMC Biophys.

[51] Agudelo-Romero P, Carbonell P, Perez-Amador MA, Elena SF (2008). Virus adaptation by manipulation of host’s gene expression. PLoS ONE.

[52] Duffy S, Shackelton LA, Holmes EC (2008). Rates of evolutionary change in viruses: patterns and determinants. Nat Rev Genet.

[53] Koonin EV, Dolja VV (2013). A virocentric perspective on the evolution of life. Curr Opin Virol.

[54] Faria NR, Suchard MA, Rambaut A, Streicker DG, Lemey P (2013). Simultaneously reconstructing viral cross-species transmission history and identifying the underlying constraints. Phil Trans R Soc B.

[55] Duffy S (2018). Why are RNA virus mutation rates so damn high?. PLoS Biol.

[56] Rohwer F, Prangishvili D, Lindell D (2009). Roles of viruses in the environment. Environ Microbiol.

[57] O ´Malley MA (2016). The ecological virus. Stud Hist Philos Biol Sci.

[58] Harris HMB, Hill C (2021). A place for viruses on the tree of life. Front Microbiol.

[59] Dupré J, O’Malley MA (2009). Varieties of living things: life at the intersection of lineage and metabolism. Philos Theor Biol.

[60] Jheeta S (2013). Final frontiers: the hunt for life elsewhere in the universe. Astrophys Space Sci.

[61] Keel WC (2007). The road to galaxy formation.

